# Evaluating the scripts and thresholds of general practitioners for diagnosing heart failure in elderly people

**DOI:** 10.1186/s12875-016-0481-4

**Published:** 2016-07-21

**Authors:** Klaartje Decaluwe, Jan Degryse, Bert Vaes

**Affiliations:** Department of Public Health and Primary Care, KU Leuven (KUL), Leuven, Belgium; Institute of Health and Society, Université catholique de Louvain (UCL), Brussels, Belgium

**Keywords:** Primary care, Heart failure, Diagnostic script, Diagnostic threshold, Aged

## Abstract

**Background:**

Multiple diagnostic algorithms for heart failure exist. However, it is unclear whether these algorithms are incorporated in the ‘scripts’ clinicians use in every day practice. Scripts are networks of organised knowledge that are acquired and accumulated during clinical training and are refined with each clinical encounter. This study was conducted to evaluate the scripts and thresholds that GPs use to diagnose heart failure in patients aged 75 years and older.

**Methods:**

The scripts and thresholds of 130 Belgian GPs in training and 63 experienced trainers were compared using an online questionnaire based on the same principles as the script concordance test. Two major cases with an open question and 19 minor cases with closed questions were presented. For the minor cases, all of the respondents were asked to assign a diagnostic power to individual cues. Based on these powers, a diagnostic threshold was calculated for each respondent for the two major cases.

**Results:**

The trainers and trainees used the same scripts to diagnose heart failure in the two major cases. Only ~50 % of the participants used natriuretic peptides in their scripts, although they judged it as the most powerful marker to demonstrate or exclude heart failure. The power that respondents gave to several cues differed significantly according to the context in which these cues were presented. In general, the average exclusive power of different cues was lower than the demonstrative power of the cues. There was no difference in diagnostic threshold between the trainers and trainees.

**Conclusion:**

Young, inexperienced GPs used the same scripts as older, more experienced GPs. In general, technical investigations were less frequently queried, compared to elements of the medical history and the clinical examination. The clinical context had a strong impact on the diagnostic power that was assigned to different factors.

**Electronic supplementary material:**

The online version of this article (doi:10.1186/s12875-016-0481-4) contains supplementary material, which is available to authorized users.

## Background

Several diagnostic algorithms for heart failure have been proposed by different national and international guidelines. These algorithms have used combinations of reported symptoms, clinical signs and technical investigations, such as blood tests, chest X-ray, electrocardiography and echocardiography, to diagnose heart failure [1–5]. In young patients, the diagnosis of heart failure is relatively straightforward. However, heart failure is more common in the elderly, and the specificity of signs and symptoms decreases drastically in these patients [6, 7]. Furthermore, the diagnosis of heart failure in older patients is often complicated by the presence of multiple comorbidities and of polypharmacy.

Two recent studies investigated the diagnostic value of signs and symptoms and additional technical examinations, such as natriuretic peptides, for the diagnosis of new-onset heart failure in elderly primary care patients, and these studies both developed clinical decision rules to help clinicians [8, 9]. Additionally, the MICE decision rule, based on a large meta-analysis of the existing literature, could also be applied in patients aged 75 years old and older [10, 11].

However, it is unclear whether these algorithms and clinical decision rules are present in the diagnostic ‘scripts’ that clinicians use in everyday clinical practice. The diagnostic process that every clinician uses is a result of scripts and thresholds. Scripts are networks of organised knowledge that are acquired and accumulated during clinical training and are refined with each clinical encounter. Clinicians mobilise these scripts to process information and progress towards solutions for difficult clinical problems [12–14]. A diagnostic threshold is a certainty that must be reached to accept or reject a diagnosis. Scripts and thresholds are known concepts in clinical reasoning [15, 16]. To date, there have been few studies that have investigated the clinical reasoning behind the diagnosing of heart failure by general practitioners (GPs) [17–21]. Moreover, previous studies have not quantified the importance of each factor in the diagnostic process. Furthermore, thresholds have not been evaluated in these studies.

Therefore, this study was conducted to investigate the scripts and thresholds that GPs use to diagnose heart failure in patients aged 75 years old and older. The scripts and thresholds of unexperienced GPs in training and of their experienced trainers were compared.

## Methods

### Participants

In this study, early career physicians and older, more experienced doctors were compared in Belgium. The early career physicians were students who finished their Master’s degrees in medical science and were in their second or third year of specialty training programs in family medicine. This group was supposed to have up-to-date theoretical knowledge and background. The older, more experienced doctors were GPs responsible for the training of these trainees. They had acquired experience and practical skills as GPs for at least 5 years. In total, 436 trainees and their trainers were contacted by mail to complete an online questionnaire. At the beginning of the questionnaire, each respondent registered the number of years that they already worked as a GP and how familiar they felt with the diagnosis of heart failure in clinical practice (Likert scale, 1 [low] to 10 [high]).

### Questionnaire

An online questionnaire was composed, based on the principles of the script concordance test [14], including two major case vignettes with an open question and 19 minor case vignettes with closed questions (see Additional file 1 for example). All of the cases presented patients aged 75 years old or older with the possibility of experiencing heart failure. In the two major case vignettes, a video interview and text were used to present the case (the stimulus format). Next, each participant was offered the opportunity to see the information on 32 possible symptoms, signs and technical investigations (the response format). After clicking on a specific cue, the participants received detailed information about that cue: the presence or absence of the sign or symptom or the result of the technical investigation was shown. The respondents were asked to click on the presented cues until they were sufficient certain about the presence or absence of heart failure. Subsequently, they were asked to indicate the degree of certainty with their diagnosis (Likert scale 1 [low] to 10 [high]). Each click and the sequence of clicks for every respondent were registered. The 32 cues were selected based on guidelines and previous epidemiological research [2, 8–11]. The first case was a patient with a high probability of heart failure, and the second case was a patient with a low probability of heart failure.

In the 19 minor cases, the power the respondents gave to individual symptoms, signs and technical investigations was explored. For every case, two cues were individually offered. For each cue, the respondent was asked to indicate how the presence and the absence (in order to calculate the demonstrative and the exclusive power) of the cue influenced the presence of heart failure in the presented case (visual scale with ‘almost excluded’ at one end and ‘almost certain’ at the other end, Likert scale 1 to 7). The cues were chosen based on the clinical decision rules from Kelder and Oudejans and the MICE rule for diagnosing heart failure [8–11]. In total, each respondent evaluated 16 different cues. Most of the cues were offered multiple times in different cases. These 16 cues were also used in the two major cases.

### Sample size calculation

Sample size calculations were primarily based on considerations in relation to the hypothesis that trainees use different scripts than trainers. Two approaches were used in order to operationalize this hypothesis and to proceed to a sample size calculation.

Firstly, the number of cues that were chosen was registered and compared between the trainers and trainees (*χ*^2^ test), for the two major cases separately. A 10 % difference was considered as meaningful and it was assumed that trainees would need 80 % of the available cues and trainers would use 50 % of the available cues to reach a threshold. This yielded a sample size of 45 in each group.

Secondly, the average diagnostic power attributed by trainees and trainers to each cue was compared in the minor cases. The power of an argument was captured on a 7-point scale. The Man-Whitney statistic was used for this purpose. Power calculations for non-parametric statistics are based on simulation [22]. It was assumed that experienced physicians would discriminate better for at least 10 % in each category. The simulation was done using a probability of a type I error of 0.05 and a power of 0.8 and was based on a rank-order table that was constructed as an expression of the assumption. This approach yielded a sample size of 49 participants in each group.

### Data analysis

All of the data were converted into numeric values and were saved in a Microsoft Excel (Microsoft Corporation, Redmond, WA, USA) file. The frequency with which each cue was chosen was registered and compared between the trainers and trainees (*χ*^2^ test), for the two major cases separately. The rank order of each cue in the diagnostic process was compared between the groups with the Mann–Whitney *U* test.

For the 19 minor cases, the power of the presence and absence of each cue ranged between 1 and 7 and was converted to: −3 = heart failure almost excluded; −2 = heart failure very unlikely; −1 = heart failure unlikely; 0 = 50 % chance that heart failure is present or absent; +1 = possible heart failure; +2 = probable heart failure; and +3 = almost certain heart failure. When the diagnostic power of a cue was scored two or more times in different contexts, Friedman’s test or Wilcoxon’s matched-pair signed-rank test was used to evaluate whether a significant difference existed. Subsequently, for each respondent, the average diagnostic power for each cue was calculated. The average power for each cue was then compared between the trainers and trainees. The frequency with which each cue was used as an exclusive cue for heart failure was also registered.

Based on the average power of each cue for each individual respondent, a diagnostic threshold for the two major cases was calculated. This threshold was the sum of the individual diagnostic powers of the cues that each respondent used in his or her own diagnostic process. A distinction was made between the cues that were used to exclude heart failure and those used to demonstrate heart failure by each respondent. The difference between the sum of the exclusive powers and the sum of the demonstrating powers for each respondent was used as the individual diagnostic threshold, which was considered a ‘proxy’ threshold because diagnostic power was not calculated for all 32 of the possible cues in the two major cases but for a selection of 16 cues, as described above. The Mann–Whitney *U* test was used to compare the diagnostic threshold between the trainers and trainees.

The statistical analyses were performed using SPSS software, version 22.0 (SPSS Inc., Chicago, IL, USA).

## Results

### Participants

In total, 436 trainees and their trainers were invited. The response rate was 22.1 % (*n* = 193) with 130 respondents (33.5 %) in the trainee group and 63 respondents (14 %) in the trainer group. The online questionnaire was answered by 28 men (21.5 %) and 102 women (78.5 %) in the trainee group and by 44 men (69.8 %) and 19 women (30.2 %) in the trainer group. The trainers had a mean clinical experience of 26.0 ± 9.5 years, and trainees had a maximum of 2 years of clinical experience. The trainers gave themselves a median score of 7 (IQR 6–9) for experience with diagnosing heart failure, while the trainees gave themselves a median score of 4 (IQR 3–5) (*P* < 0.001).

### Major cases

The first case, with a high probability of heart failure, was diagnosed positive for heart failure by 187 respondents. The remaining six (three trainees and three trainers [*χ*^2^, *P* = 0.3]) judged that it was not heart failure. Overall, the trainers were more certain of their diagnoses than the trainees (8 [IQR 6–9] vs 7 [IQR 6–8], *P* = 0.016). To reach their diagnoses, the trainees used a median of 12 (IQR 9–19) cues and the trainers 14 (IQR 10–19) cues (*P* = 0.32). Table 1 lists the frequencies with which the cues were chosen and the median rank order of each cue in the scripts that were applied. Only the item ‘dyspnoea at rest’ was chosen significantly more by the trainees than by the trainers, although the trainers chose this item more rapidly in their diagnostic processes than the trainees. For three other cues (weight loss, vertigo and abdominal ultrasound) a trend (*P* < 0.10) was seen towards more rapid usage of these items by the trainers than by trainees. However, the first five items that were most frequently used did not differ between the trainees and trainers (orthopnoea, dyspnoea at rest, history of oedema, dyspnoea on exertion and coughing). In total 41 (21.2 %) respondents (31 trainees and 10 trainers [*χ*^2^, *P* = 0.20]) did not choose any technical investigation. Only 105 (54.4 %) respondents wanted to know the level of the natriuretic peptides and 87 (45.0 %) the results of electrocardiography.

**Table 1:** The frequency cues that were used with the median rank order of each cue in the scripts that were applied for case 1

	Trainee			Trainer		
	Number of participants that clicked on the cue	Place of the cue		Number of participants that clicked on the cue	Place of the cue	
	n (%)	Range	Median (IQR)	n (%)	Range	Median (IQR)
History						
Orthopnoea	107 (82.3)	1–12	2 (1–3)	49 (77.8)	1–4	2 (2–3)
History of oedema	106 (81.5)	1–22	4 (2–6)	53 (84.1)	1–13	3 (2–5)
Dyspnoea at rest	88 (67.7)	1–16	2 (1–3)	33 (52.4)*	1–7	1 (1–2)**
Fatigue	73 (56.2)	1–21	6 (3–8)	37 (58.7)	2–17	6 (4–8)
Dyspnoea on exertion	69 (53.1)	1–17	3 (2–4)	33 (52.4)	1–14	3 (2–4)
Coughing	67 (51.5)	2–18	5 (3–6)	39 (61.9)	1–16	4 (3–6)
Retrosternal pain	63 (48.5)	1–20	5 (4–7)	35 (55.6)	1–10	5 (3–7)
Nocturnal paroxysmal dyspnoea	54 (41.5)	2–24	9 (5–13)	32 (50.8)	3–17	7 (5–11)
Palpitations	39 (30.0)	3–16	10 (8–13)	22 (34.9)	3–16	9 (6–11)
Fever	35 (26.9)	3–19	6 (5–7)	18 (28.6)	1–16	6 (5–7)
Syncope	29 (22.3)	3–13	11 (7–12)	13 (20.6)	4–14	8 (5–11)
Appetite	28 (21.5)	1–12	2 (1–5)	14 (22.2)	1–11	3 (1–6)
Weight loss	25 (19.2)	4–15	11 (9–13)	15 (23.8)	2–15	9 (5–10)***
Vertigo	20 (15.4)	2–23	9 (9–10)	10 (15.9)	4–12	8 (5–10)***
Clinical examination						
Lung auscultation	103 (79.2)	1–19	8 (6–12)	49 (77.8)	1–16	8 (6–11)
Clinical oedema	97 (74.6)	1–22	10 (6–13)	46 (73.0)	2–19	11 (7–13)
Heart auscultation	90 (69.2)	1–18	8 (5–11)	42 (66.7)	1–15	9 (7–12)
Jugular venous pressure	89 (68.5)	1–20	9 (6–13)	38 (60.3)	3–17	9 (6–11)
Weight	81 (62.3)	2–27	12 (8–17)	46 (73.0)	4–22	13 (9–15)
Parameters (e.g., pulse)	71 (54.6)	1–21	11 (7–16)	32 (50.8)	5–18	11 (9–15)
Respiratory rate and saturation	55 (42.3)	4–23	13 (8–19)	32 (50.8)	3–20	12 (9–17)
Abdominal examination	43 (33.1)	4–28	18 (11–23)	19 (30.2)	10–23	17 (14–22)
Peripheral vessels	41 (31.5)	8–26	17 (12–21)	13 (20.6)	10–21	19 (14–21)
Apex beat	38 (29.2)	7–29	20 (11–24)	20 (31.7)	5–24	16 (10–23)
Neurological examination	26 (20.0)	11–30	24 (19–26)	13 (20.6)	11–25	21 (17–25)
Technical investigation						
Chest X-ray	72 (55.4)	3–36	15 (10–23)	37 (58.7)	3–32	14 (11–23)
NT-proBNP	67 (51.5)	1–33	14 (8–23)	38 (60.3)	1–29	14 (11–20
Electrocardiography	54 (41.5)	1–28	16 (10–24)	33 (52.4)	2–26	15 (12–19)
Complete blood count and serum creatinine	39 (30.0)	7–35	17 (13–28)	26 (41.3)	9–31	19 (15–25)
Spirometry	30 (23.1)	9–37	24 (16–31)	20 (31.7)	1–33	21 (14–30)
Liver function tests	25 (19.2)	12–34	26 (20–30)	13 (20.6)	12–30	23 (18–29)
Abdominal echography	22 (16.9)	8–32	26 (18–28)	15 (23.8)	9–28	19 (17–26)***

*χ ^2^ test, *P* < 0.05

**Mann–Whitney *U* test, *P* < 0.05

***Mann–Whitney *U* test, *P* < 0.10

The second case, designed with a low probability of heart failure, was diagnosed negative for heart failure by 179 respondents. The remaining 14 (nine trainees and five trainers [*χ*^2^, *P* = 0.80]) judged that it was heart failure. No difference in diagnostic certainty was found between the trainers and trainees (median 7 [IQR 6–9] vs 7 [IQR 6–8], *P* = 0.33). The trainees used a median of 14 (IQR [9–20]) cues and the trainers a median of 16 (IQR [9–23]) cues to reach their diagnoses (*P* = 0.38). Table 2 lists the frequency and the rank order of each item. The trainers chose the items ‘weight loss’ and ‘chest X-ray’ significantly more often than the trainees. A trend (*P* < 0.10) towards different frequencies between the trainees and trainers was observed for three items: orthopnoea, peripheral vessels and abdominal examination. The rank order of the different cues in the individual diagnostic scripts was not different between the trainees and trainers. The first five items that were most frequently used did not differ between the trainees and trainers and were the same as for case 1. In total, 26 (13.5 %) respondents (20 trainees and 6 trainers [*χ*^2^, *P* = 0.26]) did not choose any technical investigations. Only 106 (54.9 %) respondents wanted to know the level of the natriuretic peptides and 93 (48.2 %) the results of electrocardiography.

**Table 2:** The frequency of cues that were used with the median rank order of each cue in the scripts that were applied for case 2

	Trainee			Trainer		
	Number of participants that clicked on the cue	Place of the cue		Number of participants that clicked on the cue	Place of the cue	
	n (%)	Range	Median (IQR)	n (%)	Range	Median (IQR)
History						
Orthopnoea	107 (82.3)	1–19	2 (2–3)	46 (73.0)**	1–6	2 (2–3)
History of oedema	106 (81.5)	1–24	5 (3–9)	43 (68.3)	1–10	6 (4–9)
Dyspnoea at rest	88 (67.7)	1–13	2 (1–3)	47 (74.6)	1–21	2 (1–2)
Fatigue	73 (56.2)	1–14	6 (5–8)	41 (65.1)	1–16	6 (5–8)
Dyspnoea on exertion	69 (53.1)	1–20	3 (2–4)	44 (69.8)	1–22	3 (2–4)
Coughing	67 (51.5)	1–24	5 (4–6)	44 (69.8)	1–9	4 (3–5)
Retrosternal pain	63 (48.5)	2–23	6 (5–7)	35 (55.6)	2–8	5 (4–7)
Nocturnal paroxysmal dyspnoea	54 (41.5)	2–27	9 (6–14)	32 (50.8)	4–14	10 (7–14)
Palpitations	39 (30.0)	2–26	9 (7–13)	26 (41.3)	4–13	10 (6–13)
Fever	35 (26.9)	2–22	6 (5–7)	21 (33.3)	3–21	6 (5–6)
Syncope	29 (22.3)	6–14	11 (10–12)	12 (19.0)	6–11	11 (10–11)
Appetite	28 (21.5)	1–10	1 (1–1)	18 (28.6)	1–7	1 (1–1)
Weight loss	25 (19.2)	1–25	10 (7–12)	35 (55.6)*	1–19	8 (6–12)
Vertigo	20 (15.4)	1–28	9 (9–11)	14 (22.2)	4–23	9 (8–9)
Clinical examination						
Lung auscultation	105 (80.8)	1–26	9 (6–13)	49 (77.8)	2–16	9 (7–13)
Heart auscultation	98 (75.4)	1–27	9 (6–13)	44 (69.8)	1–15	10 (7–14)
Clinical oedema	88 (67.7)	1–24	12 (8–18)	45 (71.4)	1–20	12 (8–17)
Peripheral vessels	73 (56.2)	2–26	14 (9–21)	27 (42.9)**	2–23	16 (13–21)
Jugular venous pressure	71 (54.6)	1–25	12 (8–17)	37 (58.7)	1–28	12 (9–17)
Parameters (e.g., pulse)	67 (51.5)	3–21	12 (9–18)	28 (44.4)	6–18	13 (11–18)
Weight	65 (50.0)	1–30	15 (10–22)	37 (58.7)	3–23	15 (12–21)
Respiratory rate and saturation	63 (48.5)	3–25	15 (11–20)	35 (55.6)	5–21	15 (11–19)
Abdominal examination	40 (30.8)	1–30	21 (15–24)	27 (42.9)**	4–25	19 (15–23)
Apex beat	35 (26.9)	7–31	24 (15–25)	22 (34.9)	2–26	19 (14–24)
Neurological examination	29 (22.3)	8–32	25 (17–26)	18 (28.6)	7–27	23 (17–25)
Technical investigation						
Chest X-ray	71 (54.6)	2–42	16 (11–25)	44 (69.8)*	1–32	16 (11–25)
NT-proBNP	71 (54.6)	2–38	15 (9–24)	35 (55.6)	1–29	19 (14–25)
Electrocardiography	58 (44.6)	5–35	19 (12–26)	35 (55.6)	6–27	18 (12–24)
Spirometry	54 (41.5)	4–43	18 (12–32)	34 (54.0)	3–33	21 (13–30)
Complete blood count and serum creatinine	49 (37.7)	6–40	20 (14–30)	27 (42.9)	3–31	19 (16–28)
Liver function tests	27 (20.8)	10–39	29 (22–30)	14 (22.2)	4–30	28 (24–29)
Abdominal echography	26 (20.0)	13–36	27 (20–28)	15 (23.8)	13–28	26 (21–27)

*χ ^2^ test, *P* < 0.05

**χ ^2^ test, *P* < 0.10

### Minor cases

The diagnostic power of 16 different cues was measured for each respondent by presenting 19 minor cases. The power that the respondents gave to several cues differed significantly according to the context in which each cue was presented (Table 3).

**Table 3:** Differences in diagnostic power of the cues in the 19 minor cases based on the clinical context

	Total group	Trainee	Trainer
Lung auscultation			
Normal^a^	*P* < 0.001	*P* = 0.003	*P* = 0.007
Basal crackles^b^	*P* < 0.001	*P* < 0.001	*P* = 0.001
Wheezing^b^	*P* < 0.001	*P* < 0.001	*P* = 0.074
Dyspnoea			
Absent^b^	*P* = 0.059	*P* = 0.51	*P* = 0.020
Present^b^	*P* = 0.58	*P* = 0.78	*P* = 0.56
NT-proBNP			
Normal^a^	*P* = 0.002	*P* = 0.098	*P* = 0.007
Increased^a^	*P* = 0.054	*P* = 0.056	*P* = 0.17
Heart murmur			
Absent^b^	*P* = 0.51	*P* = 0.70	*P* = 0.13
Present^b^	*P* = 0.25	*P* = 0.40	*P* = 0.41
Orthopnoea			
Absent^a^	*P* = 0.035	*P* = 0.14	*P* = 0.16
Present^a^	*P* < 0.001	*P* < 0.001	*P* = 0.25
Jugular venous pressure			
Normal^a^	*P* = 0.68	*P* = 0.43	*P* = 0.15
Increased^a^	*P* < 0.001	*P* < 0.001	*P* < 0.001
Nocturnal paroxysmal dyspnoea			
Absent^b^	*P* = 0.62	*P* = 0.79	*P* = 0.23
Present^b^	*P* = 0.43	*P* = 0.96	*P* = 0.17
Apex beat			
Normal^b^	*P* = 0.80	*P* = 0.85	*P* = 0.65
Displaced^b^	*P* = 0.038	*P* = 0.041	*P* = 0.41
Oedema			
Absent^a^	*P* = 0.20	*P* = 0.27	*P* = 0.32
Present^a^	*P* < 0.001	*P* < 0.001	*P* = 0.054
History myocardial infarct			
Absent^b^	P = 0.69	*P* = 0.70	*P* = 0.90
Present^b^	*P* = 0.32	*P* = 0.58	*P* = 0.35
Electrocardiography			
Normal^b^	*P* = 0.024	*P* = 0.063	*P* = 0.20
Abnormal^b^	*P* < 0.001	*P* < 0.001	*P* = 0.69
Chest X-ray (cardiothoracic index)			
Normal^b^	*P* = 0.048	*P* = 0.070	*P* = 0.41
Abnormal^b^	*P* < 0.001	*P* < 0.001	*P* < 0.001
Pulse rate			
Regular^b^	*P* < 0.001	*P* < 0.001	*P* = 0.007
Irregular^b^	*P* = 0.17	*P* = 0.68	*P* = 0.002
Appetite			
Normal^b^	*P* = 0.58	*P* = 0.23	*P* = 0.32
Decreased^b^	*P* < 0.001	*P* < 0.001	*P* = 0.43

^a^Difference in distribution of the given power between the cases (>2) with Friedman’s test

^b^Difference in distribution of the given power between the cases (≤2) with Wilcoxon’s matched-pair signed-rank test

Table 4 shows the average diagnostic power of each cue. The average powers were compared between the trainees and trainers. A normal level of NT-proBNP and the absence of dyspnoea were scored as the strongest cues to exclude heart failure, while an increased level of NT-proBNP, the presence of orthopnoea and a history of a myocardial infarction were chosen as the strongest cues to demonstrate heart failure. In general, the average exclusive power of the different cues was lower than the demonstrative power of the cues that were presented.

**Table 4:** Diagnostic power of the individual cues in the 19 minor cases

	Total group	Trainee		Trainer	
	Average power, median (IQR)	Average power, median (IQR)	Cue used to exclude HF n (%)	Average power, median (IQR)	Cue used to exclude HF n (%)
Lung auscultation					
Normal	−0.5 (−1.0, −0.25)	−0.50 (−1.0, −0.20)	98 (75.4)	−0.75 (−1.0, −0.25)	54 (85.7)
Basal crepitation	1.5 (1.0, 2.0)	1.5 (1.5, 2.0)	1 (0.8)	1.5 (1.0, 2.0)	1 (1.6)
Wheezing	0 (−1.0, 0.50)	−0.50 (−1.0, 0.13)	72 (55.4)	0 (−0.50, 0.50)*	17 (27.0)
Dyspnoea					
Absent	−1.0 (−1.5, −0.50)	−1.0 (−1.0, −0.50)	114 (87.7)	−1.0 (−1.5, −0.50)	58 (92.1)
Present	1.5 (1.0, 2.0)	1.5 (1.0, 2.0)	2 (1.5)	1.5 (1.0, 2.0)	0 (0)
NT-proBNP					
Normal	−1.3 (−2.3, −0.67)	−1.3 (−2.3, 0.67)	113 (86.9)	−1.3 (−2.7, −0.67)	53 (84.1)
Increased	2.0 (1.3, 2.7)	2.0 (1.3, 2.7)	0 (0)	2.0 (1.3, 2.3)	0 (0)
Heart murmur					
Absent	0 (−0.50, 0)	0 (−0.50, 0)	42 (32.3)	0 (−0.50, 0)	28 (44.4)
Present	1.0 (0.50, 1.5)	1.0 (0.50, 1.5)	2 (1.5)	1.0 (0.50, 1.5)	2 (3.2)
Orthopnoea					
Absent	−0.67 (−1.0, −0.33)	−0.67 (−1.0, −0.33)	101 (77.7)	−0.67 (−1.0, −0.33)	50 (79.4)
Present	2.0 (1.7, 2.7)	2.0 (1.7, 2.7)	0 (0)	2.0 (1.7, 2.7)	0 (0)
Jugular venous pressure					
Normal	−0.33 (−1.0, 0)	−0.33 (−0.67, 0)	66 (50.8)	−0.67 (−1.0, 0)*	42 (66.7)
Increased	1.7 (1.3, 2.3)	1.7 (1.3, 2.1)	0 (0)	1.7 (1.0, 2.3)	0 (0)
Nocturnal paroxysmal dyspnoea					
Absent	−0.5 (−1.0, 0)	−0.50 (−1.0, 0)	84 (64.6)	−0.50 (−1.0, 0)	43 (68.3)
Present	1.0 (1.0, 2.0)	1.0 (0.50, 1.6)	2 (1.5)	1.5 (1.0, 2.0)*	1 (1.6)
Apex beat					
Normal	0 (0, 0)	0 (0, 0)	19 (14.6)	0 (−0.50, 0)*	19 (30.2)
Displaced	1.0 (1.0, 1.5)	1.0 (1.0, 1.5)	1 (0.8)	1.0 (1.0, 1.5)*	0 (0)
Oedema					
Absent	−0.75 (−1.0, 0)	−0.50 (−1.0, 0)	96 (73.8)	−0.75 (−1.0, 0)	47 (74.6)
Present	1.5 (1.3, 2.0)	1.8 (1.3, 2.0)	0 (0)	1.5 (1.3, 2.0)	0 (0)
History myocardial infarct					
Absent	−0.50 (−0.75, 0)	−0.50 (−0.63, 0)	71 (54.6)	0 (−1.0, 0)	29 (46.0)
Present	2.0 (1.0, 2.0)	2.0 (1.0, 2.0)	0 (0)	1.5 (1.0, 2.0)	0 (0)
Electrocardiography					
Normal	0 (−1, 0)	0 (−1.0, 0)	61 (46.9)	−0.50 (−1.0, 0)	34 (54.0)
Abnormal	0.50 (0, 1.0)	0.50 (0, 1.0)	4 (3.1)	0.50 (0, 1.0)	5 (7.9)
Chest X-ray (cardiothoracic index)					
Normal	−0.50 (−1.0, 0)	−0.50 (−1.0, 0)	78 (60.0)	−0.50 (−1.0, 0)	39 (61.9)
Abnormal	1.5 (1.0, 2.0)	1.5 (1.0, 2.0)	1 (0.8)	1.5 (1.0, 2.0)	0 (0)
Pulse rate					
Regular	0 (−0.50, 0)	0 (0, 0)	30 (23.1)	0 (−0.50, 0)	21 (33.3)
Irregular	1.0 (1.0, 1.8)	1.0 (1.0, 1.5)	6 (4.6)	1.5 (1.0, 2.0)*	0 (0)
Appetite					
Normal	0 (0, 0)	0 (0, 0)	15 (11.5)	0 (0, 0)	11 (17.5)
Decreased	0.50 (0, 1.0)	0.50 (0, 1.0)	10 (7.7)	1.0 (0, 1.0)*	3 (4.8)

*Significant difference in mean power assigned to the cues in the minor cases between trainees and trainers (Mann–Whitney *U* test, *P* < 0.05)

Body mass index (BMI) and pulse rate were offered several times with different cut-off values: the diagnostic powers of a BMI of 28 kg/m^2^, 24.8 kg/m^2^, 19.5 kg/m^2^ and 18 kg/m^2^ were not different between the trainees and trainers and were on average scored as a neutral cue (average power = 0); the diagnostic powers of pulse rates of 66/min and 109/min were scored differently between the trainees and trainers (0 [IQR 0–0] vs 0 [−1.0–0], *P* = 0.001 and 1.0 [0–1.0] vs 1.0 [1.0–2.0], *P* < 0.001, respectively).

### Diagnostic threshold

**Fig. 1:**
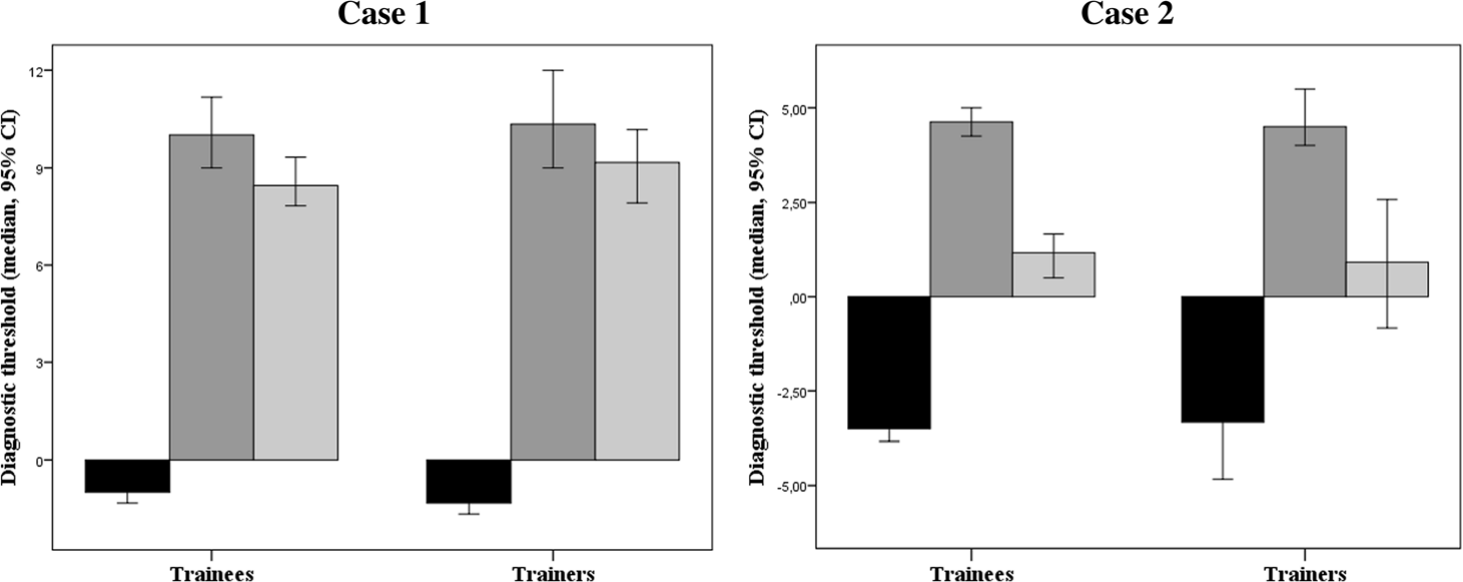
Threshold for the diagnosis of heart failure for trainers and trainees. Exclusive power  Demonstrating power  Diagnostic threshold

Figure 1 shows the exclusive power and demonstrative power and the proxy diagnostic threshold for both major cases. For the first case, the diagnostic threshold ranged between −0.17 and 23.2, with a median threshold of 8.5 (IQR 5.8–11.6) for the trainees and 9.2 (IQR 6.0–11.7) for the trainers (*P* = 0.54). For the second case, the diagnostic threshold ranged between −12.8 and 8.8, with a median threshold of 1.2 (IQR −1.2–2.9) for the trainees and 0.92 (IQR −2.5–4.0) for the trainers (*P* = 0.71). The correlation coefficient between the calculated diagnostic threshold and the diagnostic certainty that every respondent had to score was 0.24 (*P* = 0.001) for the first case and −0.14 (*P* = 0.054) for the second case.

## Discussion

This study, based on the same principles as the script concordance test, evaluated the scripts and thresholds of general practitioners in diagnosing heart failure in elderly people. No differences were identified between the trainees and trainers in the scripts that were used. In general, technical investigations were less frequently queried, compared to elements of the medical history and the clinical examination. The power that the respondents in both groups gave to several cues differed significantly according to the context in which each cue was presented. Furthermore, the assigned exclusive power of different cues was lower than the assigned demonstrative power of the cues that were presented. The proxy diagnostic threshold was the same for the trainers and trainees, but the variation between individual participants was large.

The hypothesis that more experienced clinicians would use clinical information in a more targeted manner, would use fewer cues to reach diagnostic certainty and would have different scripts than less experienced clinicians could not be confirmed. The previous literature about scripts has stated that scripts change when more experience is gained [12, 13]. A possible explanation for the discrepancy with the current findings could be that the trainers completed the questionnaire as teachers rather than as clinicians and thus clicked more cues than they would have in real life. In contrast, other studies examining the diagnosis of a difficult clinical problem, e.g., heart failure or pulmonary embolism, have supported the conclusion that there is no difference between the diagnostic strategies used by medical students and more experienced clinicians, although the variation between individual participants is large [18, 19, 23]. Possibly, the expected evolution in scripts when gaining clinical experience from a theoretical point of view is not represented in real-life clinical practice for severe diagnoses. Future research using an incomplete format design could be undertaken to investigate further the differences in diagnostic scripts based on clinical experience.

Early diagnosis of heart failure is important to initiate treatment in a timely manner to delay progression to overt heart failure [2]. However, several studies have reported the poor validity of GPs’ diagnoses, reporting both under- and over-diagnoses [24–26]. This finding could be explained by the non-specific nature of heart failure symptoms and signs, especially in older persons [9, 24, 26] and by the observation that GPs underuse objective cardiac measurements such as natriuretic peptides and echocardiography [26–28]. In heart failure guidelines [1–5], normal electrocardiography has been incorporated as a good test to exclude heart failure. The current study, however, showed that the respondents attributed little excluding power to this test, and only half of the respondents had this test included in their scripts. The overall observation in the current study that technical investigations were seldom used has also been seen in previous research. Skånér et al. observed the same trend in which no information about echocardiography was utilised by one third of the GPs in the presented situations, and only 20 % used the information about normal electrocardiography as a cue to exclude heart failure [21]. Furthermore, Skånér et al. found that GPs estimated that information on the presence of dyspnoea, a history of myocardial infarction and enlargement of the heart influenced their judgement on the presence of heart failure the most [18]. In the current study, the same trend was observed, but the respondents also individually assessed the diagnostic power of each cue. A normal level of NT-proBNP and the absence of dyspnoea were scored as the strongest cues for excluding heart failure, and an increased level of NT-proBNP, the presence of orthopnoea and the history of a myocardial infarction were chosen as the strongest cues to demonstrate heart failure. The observation that NT-proBNP was present in only ~50 % of the diagnostic strategies despite having been assigned strong diagnostic power could be explained by these tests not being reimbursed in Belgium, although these tests were incorporated into the national guidelines of 2011. Skånér et al. did not examine NT-proBNP because it was not available at that time.

The observation that GPs gave different powers to the same cues in different contexts is an addition to the existing epidemiological data, in which only one likelihood ratio for each cue is calculated. These findings could be explained by epidemiological powers being measurements, whereas the current study concerned clinical judgements based on the integration of rich contextual information. Thus, in everyday clinical practice, GPs do not use one fixed diagnostic power for different cues. This fact must be considered when estimating diagnostic thresholds.

In a low prevalence context, such as general practice, diagnostic strategies are in general more focused on finding cues that exclude a diagnosis rather than on cues that demonstrate a diagnosis. In the current study, however, the diagnostic power of different cues to exclude heart failure was on average weighted less than the diagnostic power to demonstrate heart failure. Furthermore, no differences in diagnostic strategies were observed between case 1, with a high pretest probability, and case 2, with a low pretest probability. The dominant strategy seemed to be looking for cues with demonstrative power to reach a threshold for a positive diagnosis, which could explain the rather high number of cues that were queried in both cases, although the latter could also be explained by GPs being accustomed to looking at patients in a holistic manner and thinking less in terms of algorithms. Furthermore, clinicians are most likely aware of the decreasing sensitivity and specificity of signs and symptoms for heart failure in the elderly. Some of our findings also fit well with what has been described as the ‘Acceptable Regret Approach’ [29]. When acceptable regret (regret that a physician finds tolerable upon making a wrong decision) is considered, doctors tend to order diagnostic tests at a higher level of pretest probability of disease than expected. Moreover, the proposed clinical decision rules for the diagnosis of heart failure in the elderly are also more oriented towards summing up demonstrative cues rather than exclusive cues and thus pushing clinicians more in the direction of demonstrating heart failure [8, 9]. In contrast, the current findings also call for further implementation of existing strong excluders, such as natriuretic peptides, in daily practice and further education of clinicians in using these technical investigations for the diagnosis of heart failure.

This study was the first that evaluated scripts and thresholds of GPs for diagnosing heart failure in elderly people. A strength of this study lays in each respondent being asked to score the diagnostic power of individual cues in a direct manner, compared to previous research, which only deducted the weight of each cue depending on the answers about the presence of heart failure. Furthermore, this study was the first that calculated a diagnostic threshold for the presence or absence of heart failure.

A few limitations should be considered. Firstly, the cases in the questionnaire were all fictional ‘paper’ cases with the possibility of heart failure. It is possible that these cases were less representative of real-life patients, but they all had high ‘face validity’. Furthermore, it was also important for this study to examine the strategies of different doctors to the same stimulus, rather than to examine their reactions in real situations. Secondly, the response rate of 22 % was mediocre, but this study was designed to compare diagnostic strategies between young and more experienced clinicians and was not designed to be representative of Belgian GPs. Moreover, the sampling of experienced GPs might have been a potential problem, because GPs with a special interest in training and education were selected, most likely making this group more homogeneous than a random sample of experienced GPs would have been. Thirdly, the diagnostic threshold was only a proxy threshold because not all 32 of the cues from the major cases were used, although the 16 most important cues in the literature were included. Fourthly, based on the sample size calculation a type II error could not be completely excluded, i.e., increasing in particular the number of experienced physicians in the design might have yielded a small significant difference, however this would never have triggered a clinical meaningful difference.

## Conclusion

Young and unexperienced GPs used the same diagnostic reasoning and scripts as older, more experienced GPs to diagnose heart failure in elderly people. In general, technical investigations were less frequently queried than elements of the medical history and the clinical examination. The power that the respondents in both groups gave to several cues differed significantly according to the context in which each cue was presented. Furthermore, the assigned exclusive power of different cues was lower than the assigned demonstrative power of the cues that were presented.

## Abbreviations

BMI, body mass index; GP, general practitioner; IQR, interquartile range; NT-proBNP, N terminal pro B-type natriuretic peptide

## Additional file

Additional file 1: Supplementary material. File that contains an example of a major case and an example of a minor case. (DOC 398 kb)
