# Supplementary figures and images for: Evaluating the scripts and thresholds of general practitioners for diagnosing heart failure in elderly people
Source: BMC Fam Pract. 2016 Jul 21;17:86. doi: 10.1186/s12875-016-0481-4 (PMC4955166; doi:10.1186/s12875-016-0481-4)

**Supplementary Material**

*Example of major case:*


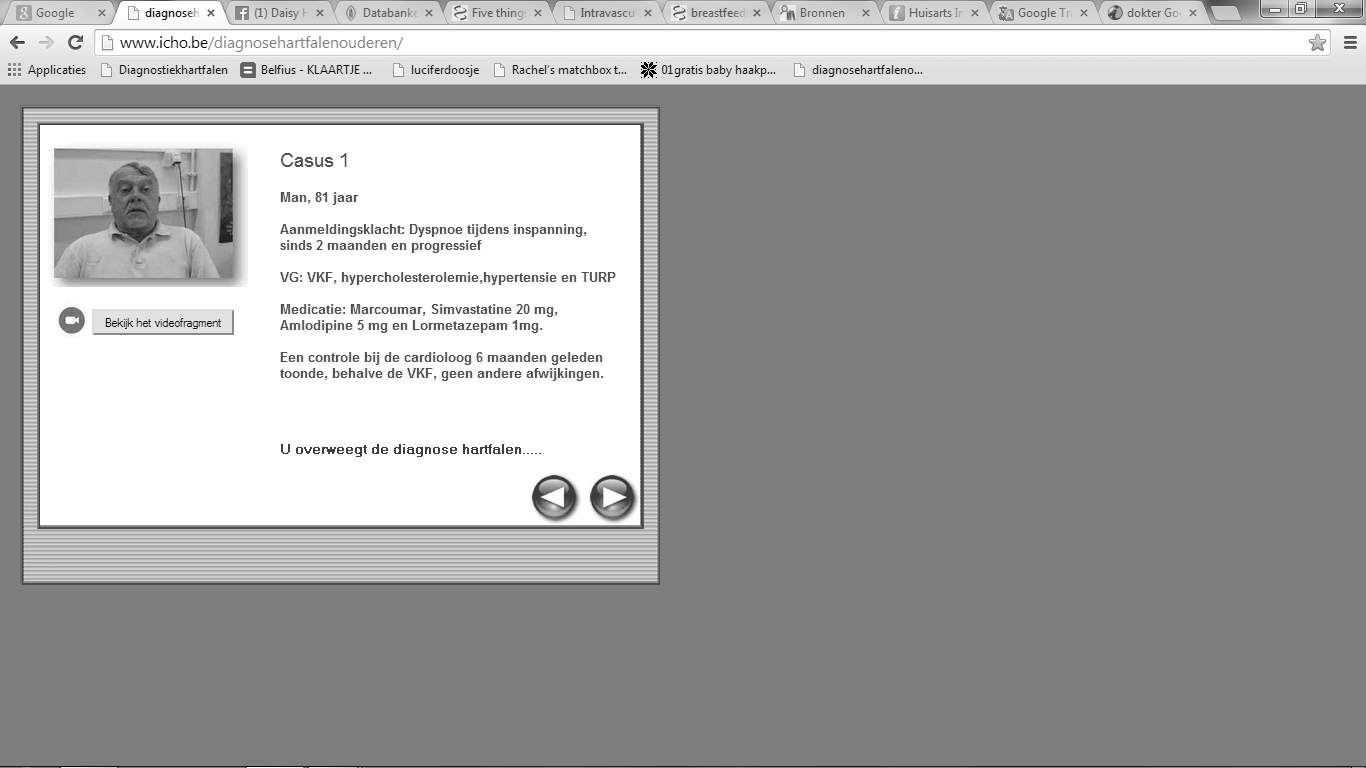


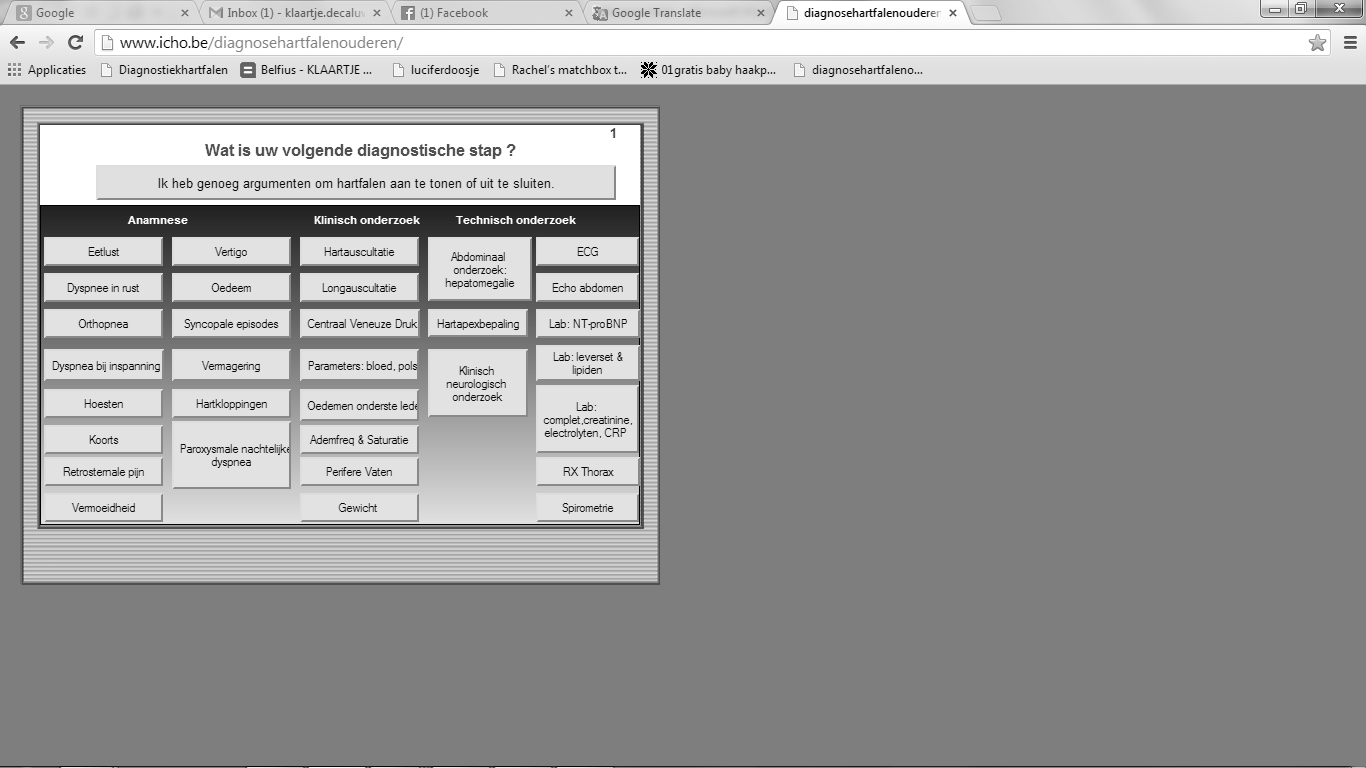


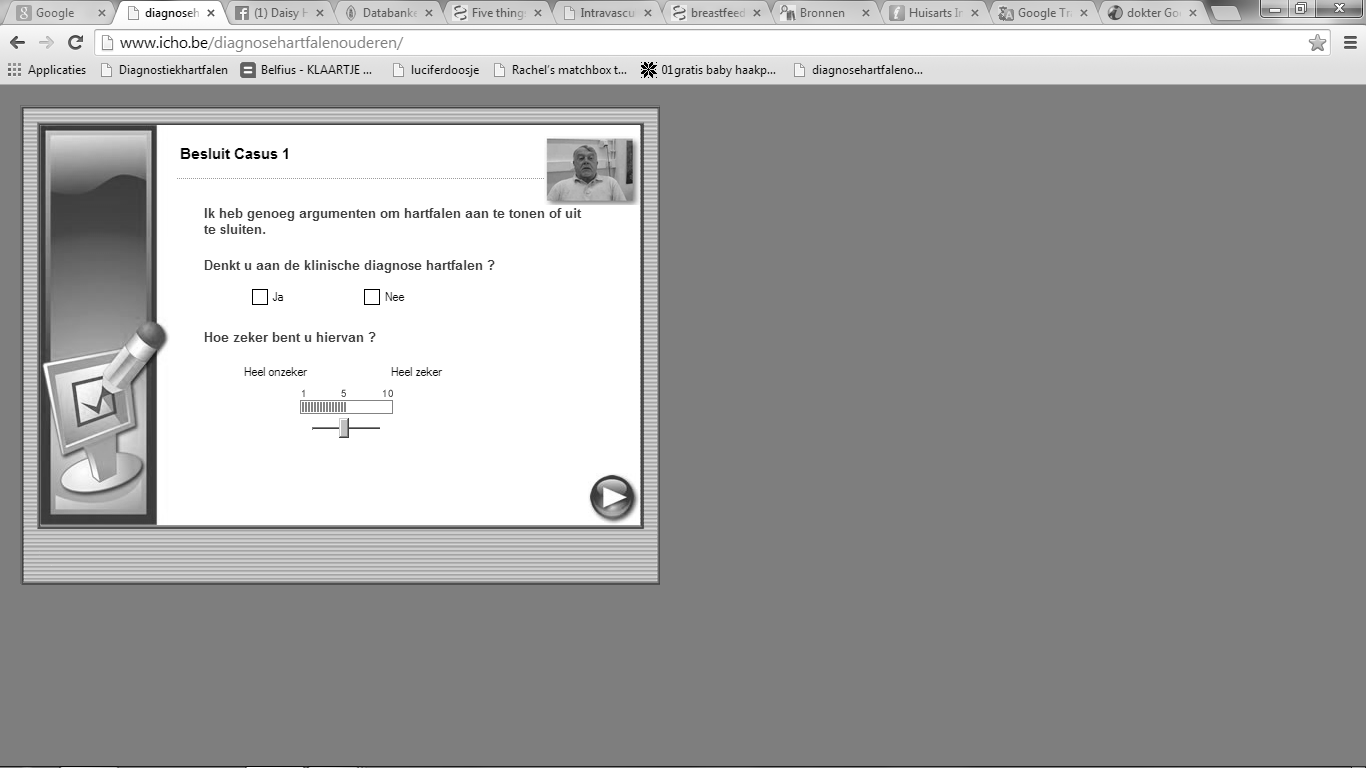


*Example of minor case:*


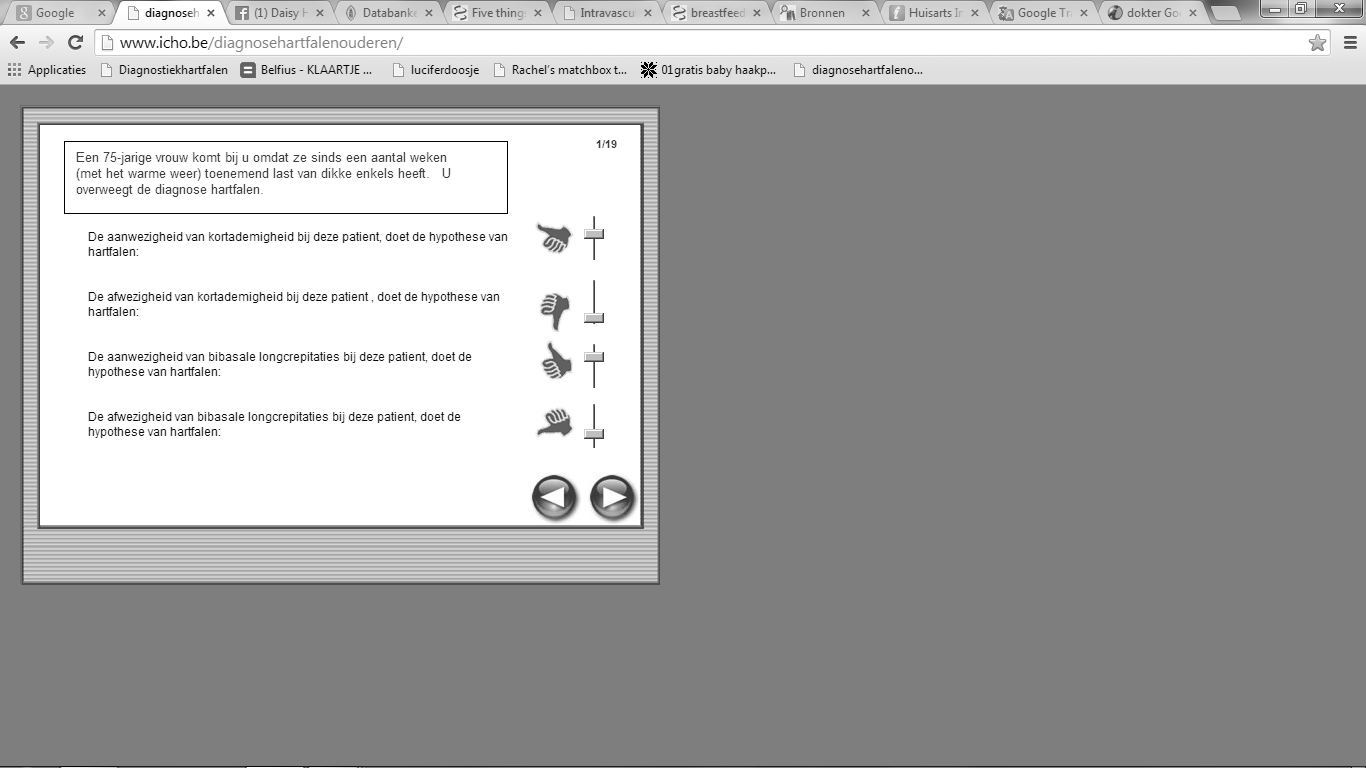

Supplement: Supplementary file 1 — Supplementary material. File that contains an example of a major case and an example of a minor case. (DOC 398 kb) [file 12875_2016_481_MOESM1_ESM.doc]
